# VQF-Based Decoupled Navigation Architecture for High-Curvature Maneuvering of Underwater Vehicles

**DOI:** 10.3390/s26030814

**Published:** 2026-01-26

**Authors:** Bowei Cui, Yu Lu, Lei Zhang, Fengluo Chen, Bingchen Liang, Peng Yao, Xiaokai Mu, Shimin Yu

**Affiliations:** 1 The College of Engineering, Ocean University of China, Qingdao 266100, China; 2 China Merchants Marine and Offshore Research Institute Co., Ltd., Shenzhen 518067, China; luyu7@cmhk.com (Y.L.);; 3 College of Shipbuilding Engineering, Harbin Engineering University, Harbin 150009, China

**Keywords:** underwater navigation, IMU/DVL integration, decoupled estimation, adaptive Kalman filter, VQF-EKF

## Abstract

To mitigate the position divergence resulting from attitude error amplification in conventional fully coupled architectures, this study proposes a decoupled navigation architecture based on the Versatile Quaternion-based Filter (VQF). This architecture removes attitude estimation from the state vector, forming a two-layer structure comprising an independent attitude module and a navigation filter. The VQF is integrated as a standalone attitude module via a standardized interface. An uncertainty quantification model is developed by extracting the VQF’s internal correction states, which maps deviations among intermediate quaternion values to a measurable uncertainty metric. To compensate for the loss of cross-covariance induced by decoupling, a dual-layer compensation mechanism is introduced: a base layer adjusts the overall uncertainty using innovation statistics, while a compensation layer explicitly propagates attitude uncertainty through parameterized noise matrices. Experimental results demonstrate that the proposed method achieves notable improvements in positioning accuracy and significantly suppresses extreme errors in high-curvature scenarios. The approach is particularly effective for high-curvature, high-dynamic applications where process noise modeling is inherently difficult. Compared to traditional fully coupled architectures, the decoupled architecture offers enhanced robustness. The complementary characteristics identified between the two architectures provide valuable insights for expanding the operational envelope of underwater navigation systems.

## 1. Introduction

Autonomous underwater vehicles (AUVs) and remotely operated vehicles (ROVs) play increasingly critical roles in ocean resource exploration, underwater structure inspection, and seabed pipeline monitoring [1]. Modern underwater operations require vehicles to execute high-curvature and variable-curvature trajectories frequently. The spatial constraints and precision requirements of these missions render straight-line or large-radius cruising inadequate. Typical scenarios include: curved trajectory tracking during seabed pipeline inspection requires close adherence to pipeline contours [2]; structural inspection of offshore oil and gas platforms requires ROVs to circumnavigate cylindrical structures [3]; vehicles equipped with manipulators must approach targets along curved paths [4]. Typical torpedo-shaped AUVs face significant maneuvering constraints due to hydrodynamic characteristics and control surface limitations [5,6], with minimum turning radii often several times their body length.When operating in confined spaces or around complex structures, vehicles must execute high-curvature trajectories, posing considerable challenges for navigation system performance.

Underwater navigation technology faces severe environmental constraints: GPS signals cannot penetrate seawater [7], and acoustic positioning systems offer limited coverage at high cost [8]. The integration of inertial measurement units (IMUs) and Doppler velocity logs (DVLs), leveraging their autonomy and continuity, constitutes the foundation of underwater navigation systems [9]. IMU/DVL integrated navigation systems perform well during straight-line cruising but experience severe accuracy degradation when executing high-curvature trajectories. High-dynamic maneuvers generate significant centripetal acceleration, which contaminates accelerometer measurements of the gravity vector [10]. During high-curvature motion, the accelerometer measures both gravitational and dynamic accelerations simultaneously, degrading attitude estimation accuracy and subsequently affecting velocity and position solutions through coordinate transformation. The root cause of this phenomenon lies in the fully coupled architecture design of traditional navigation systems.

The Kalman filtering framework widely adopted in engineering practice unifies attitude, velocity, position, and sensor biases into a 15–16 dimensional state vector, forming a fully coupled system [11]. Within this structure, off-diagonal elements of the state transition matrix form coupling terms, causing attitude errors to affect velocity and position estimates through complex coupling relationships that become significantly amplified during high-curvature motion. According to classical inertial navigation error propagation theory [12], position errors induced by gyroscope bias exhibit quadratic growth with time during straight-line motion, whereas during curved motion, attitude errors couple to velocity estimates through centripetal acceleration, exhibiting cubic growth characteristics [13]. This implies that during a 10-min high-curvature mission, position error growth rates may exceed straight-line cruising rates by more than tenfold, severely constraining operational duration.

Researchers have proposed various solutions. Motion mode adaptive methods [14] optimize navigation performance through motion pattern recognition, yet these approaches face two core limitations: first, a fundamental contradiction exists between model library size and computational complexity—as the number of modes increases, real-time performance becomes increasingly difficult to guarantee; second, mode switching introduces delays that easily generate transient errors during rapid maneuvers, and coverage of unknown complex trajectories often proves inadequate. Parameter adaptive adjustment methods [15,16] respond to motion changes through dynamic adjustment of filter parameters, but noise covariance matrix estimation typically requires 5–10 min of convergence time. In high-dynamic scenarios, error growth rates far exceed parameter adjustment speeds, resulting in methods that enhance adaptability to environmental changes yet exhibit slow response and lack predictive capability. External constraint methods [17,18] demonstrate significant effectiveness in terrestrial navigation, benefiting from abundant prior information in terrestrial environments such as road networks and building elevations. However, underwater environments lack reliable prior information [19]: seafloor terrain maps possess low resolution, ocean currents cause dynamic environmental changes, and stable feature points are scarce, rendering constraint methods difficult to apply. These methods share a common limitation: they attempt to mitigate accuracy degradation through algorithmic optimization within traditional fully coupled architectures without fundamentally altering attitude error propagation paths.

Decomposing high-dimensional systems into independent subsystems can significantly improve navigation performance and fault tolerance [20]. Lyu et al. demonstrated in AUV integrated navigation systems that an adaptive federated filter architecture reduced position error by 66% compared to fully coupled approaches, validated through 30 experimental trials with SINS/DVL/TAN integration. This empirical evidence inspires a new improvement approach: extracting independent estimation modules from traditional fully coupled navigation systems to enhance estimation accuracy through reduced coupling complexity. Huang et al. [21] achieved partial decoupling by expanding quaternions into state variables, improving attitude estimation accuracy by 15% in underwater glider applications. However, quaternions remained estimated quantities, attitude-velocity coupling terms persisted in the state transition matrix, and error amplification problems continued during high-curvature motion. Shaukat et al. [22] employed RBF neural networks as assistance, learning attitude error patterns to improve estimation, yet retained the full-state Kalman framework with neural networks serving as compensation rather than architectural reconstruction. The common limitation of these methods lies in attitude estimation remaining coupled with the navigation filter, unable to fundamentally block attitude error propagation to velocity and position through the state transition matrix.

Completely eliminating attitude-velocity coupling terms from state transition equations requires separating attitude estimation from the navigation filter and establishing an independent attitude estimation module. This requires attitude algorithms to possess three key characteristics: independent operation capability, high-precision output, and uncertainty quantification. The Versatile Quaternion-based Filter (VQF) by Laidig and Seel [23] provides an ideal solution for decoupled architectures. Compared to traditional attitude algorithms, VQF offers significant advantages: relative to EKF-AHRS, VQF eliminates the need for complex noise covariance matrix tuning and reduces computational complexity by approximately 60%; relative to complementary filters such as Madgwick and Mahony, VQF can output attitude uncertainty, providing crucial information for subsequent compensation mechanisms; VQF’s magnetic disturbance rejection capability renders it particularly suitable for underwater environments with ferromagnetic structures. VQF’s triple output satisfies the core requirements of decoupled architectures: attitude quaternions for coordinate transformations, relative acceleration for motion intensity perception and parameter adaptation, and attitude uncertainty for quantifying estimation reliability and compensating for covariance information loss caused by decoupling.

This study proposes a VQF-based decoupled navigation architecture that addresses accuracy degradation during high-curvature motion from both architectural and algorithmic perspectives. The main contributions of this research are as follows:

1. VQF-based attitude-navigation decoupling scheme: Attitude estimation is completely separated from the navigation filter state vector, with VQF serving as an independent attitude module. The attitude-velocity coupling term $F_{v\theta }$ in the state transition matrix is eliminated, blocking deterministic error propagation under high-curvature conditions.
2. Uncertainty extraction method from VQF internal states: A method is proposed to quantify attitude uncertainty through statistical deviations among quaternions at different correction stages during VQF processing, providing explicit attitude reliability metrics for the decoupled architecture.
3. Dual-layer compensation mechanism for attitude uncertainty: A noise covariance adjustment strategy is designed that combines base-layer innovation adaptation with compensation-layer attitude uncertainty parameterization. Attitude uncertainty is mapped to process and observation noise increments through kinematic coupling relationships.

The remainder of this paper is organized as follows: Section 2 establishes the theoretical foundation, analyzes degradation of traditional architectures in specific scenarios, and reveals the necessity of decoupling; Section 3 details the conditions that decoupling must satisfy; Section 4 explains the specific decoupled system design; Section 5 validates method performance through real datasets; Section 6 summarizes the work and provides future research directions.

## 2. Error Propagation Mechanisms During High-Curvature Motion

Traditional fully coupled error-state extended Kalman filters (ESKFs) exhibit significant accuracy degradation during high-curvature maneuvers involving high angular velocity turns. This chapter analyzes the fundamental causes of this phenomenon—namely, the roles of the coupling term $F_{v\theta }$ and the cross-covariance submatrix $P_{\theta v}$ in propagating attitude errors and uncertainty to velocity errors—revealing the necessity of decoupling in such scenarios.

### 2.1. High-Curvature Motion and Coupling Term Error Amplification

We first clarify the terminology. In this work, *high-curvature motion* refers to maneuvering segments with curvature κ≥0.06 $m^{-1}$ (turning radius R≤17 m). This threshold encompasses typical underwater close-proximity operations where turning radii commonly range from 5 to 20 m, while excluding near-straight-line cruising. Under such conditions, the centripetal acceleration $a_{c}=v^{2}\kappa$ becomes significant. It is $a_{c}$, rather than κ alone, that directly amplifies the coupling term $∥F_{v\theta }∥$; therefore, we focus on centripetal acceleration as the primary physical quantity in error analysis. Throughout this paper, $a_{c}$ and *g* denote scalar magnitudes (in m/$s^{2}$).

The state vector of a fully coupled IMU/DVL navigation system includes attitude, velocity, position, and biases:

(1) $$x={\left[\delta p^{T},\delta v^{T},\delta \theta^{T},\delta b_{g}^{T},\delta b_{a}^{T}\right]}^{T}\in R^{15}$$

where each component $\in R^{3}$, and δθ relates to quaternion error via $\delta q\approx {\left[1,\frac{1}{2}\delta \theta^{T}\right]}^{T}$. The error dynamics equation is [12]:

(2) $$\delta \dot{v}=F_{v\theta }\delta \theta +F_{vv}\delta vs.-C_{b}^{n}\delta b_{a}+w_{a}$$

where $w_{a}$ denotes accelerometer noise, and $F_{v\theta }=-C_{b}^{n}{\left[f_{\mathrm{imu}}\right]}_{\times }$ represents the attitude-velocity coupling term, ${\left[f_{\mathrm{imu}}\right]}_{\times }$ denotes its skew-symmetric matrix, reflecting the intensity of attitude error δθ’s influence on velocity error rate $\delta \dot{v}$. During stable cruising, $∥f_{\mathrm{imu}}∥\approx g\approx 9.8$ m/$s^{2}$ remains relatively constant, resulting in a basically constant velocity error growth rate for a given attitude error, making error propagation characteristics predictable.

High-curvature motion generates centripetal acceleration $a_{c}$, causing IMU measurements to include motion acceleration components. Taking horizontal circular motion as an example, based on the analysis by Borodacz and Szczepanski [13], the spectral norm of the coupling term satisfies:

(3) $$∥F_{v\theta }{∥}_{2}=∥f_{\mathrm{imu}}∥=\sqrt{g^{2}+a_{c}^{2}}$$

More generally, high-curvature maneuvers cause $∥f_{\mathrm{imu}}∥$ to deviate significantly from the nominal value *g*, with the magnitude of increase related to centripetal acceleration.

This implies that the same attitude error δθ produces a larger velocity error growth rate under high curvature, and that the error propagation path is amplified. Simultaneously, centripetal acceleration contaminates the accelerometer’s observation of the gravity direction [24], degrading attitude estimation accuracy itself and increasing the error source. Therefore, both increased attitude error sources and enhanced error coupling jointly lead to accumulated estimation errors in high-curvature scenarios.

### 2.2. Cross-Covariance Underestimation Mechanism

Beyond deterministic error propagation through $F_{v\theta }$, the Kalman filter relies on the cross-covariance $P_{\theta v}$ to compute correction gains that compensate for attitude errors using DVL velocity observations. However, under high-curvature conditions, $P_{\theta v}$ becomes systematically underestimated, causing the correction mechanism to fail progressively.

The attitude-velocity cross-covariance propagates according to:

(4) $$P_{\theta v}\left(k+1|k\right)=F_{\theta \theta }P_{\theta v}\left(k|k\right)F_{vv}^{⊤}+F_{\theta \theta }P_{\theta \theta }\left(k|k\right)F_{v\theta }^{⊤}+Q_{\theta v}$$

Here, $F_{\theta \theta }=\partial \dot{\theta }/\partial \theta$ and $F_{vv}=\partial \dot{v}/\partial v$ represent the attitude and velocity self-transition submatrices, characterizing how each state evolves independently within a single time step. The coupling submatrix $F_{v\theta }=\partial \dot{v}/\partial \theta$ quantifies attitude-to-velocity error propagation, as analyzed in Section 2.1. The reverse coupling $F_{\theta v}=\partial \dot{\theta }/\partial v$ vanishes identically because velocity does not appear in attitude kinematics—this asymmetry is precisely what enables attitude to be estimated independently of velocity errors in the decoupled architecture proposed herein.

The process noise terms capture stochastic uncertainty: $Q_{\theta \theta }=\sigma_{g}^{2}\Delta t\cdot I$ represents attitude process noise arising from gyroscope angular random walk, while $Q_{\theta v}=\mathrm{Cov}\left(w_{\theta },w_{v}\right)\approx 0$ since gyroscope and accelerometer noise sources are statistically independent. Under first-order approximations ($F_{\theta \theta }\approx I$, $F_{vv}\approx I$), the propagation simplifies to:

(5) $$P_{\theta v}\left(k+1|k\right)\approx P_{\theta v}\left(k|k\right)+P_{\theta \theta }\left(k|k\right)\cdot {\left(F_{v\theta }\Delta t\right)}^{⊤}$$

This result reveals that $P_{\theta v}$ growth depends on two factors: $P_{\theta \theta }$ as the uncertainty source, and $F_{v\theta }$ as the amplification coefficient. Since $P_{\theta \theta }$ increments by $Q_{\theta \theta }$ at each prediction step, underestimation of $Q_{\theta \theta }$ propagates directly to $P_{\theta v}$, with the effect amplified by $F_{v\theta }$.

Under high-curvature conditions, actual process noise increases substantially due to model violations (centripetal acceleration invalidates quasi-static assumptions), linearization errors (large attitude rates), and unmodeled dynamics (sideslip effects). The *Q* matrix, typically tuned for nominal straight-line conditions, cannot adapt in real-time [15], causing systematic underestimation of $Q_{\theta \theta }$. This underestimation originates fundamentally from observability dimension mismatch during EKF linearization [25], whereby the filter extracts spurious information from unobservable directions. Combined with the amplified $∥F_{v\theta }∥$ established in Section 2.1, the underestimation propagates from $P_{\theta \theta }$ to $P_{\theta v}$ with increasing severity, ultimately distorting the Kalman gain used for attitude correction.

### 2.3. Correction Failure and Adverse Cycles

During DVL observation updates, Kalman filtering uses velocity residuals to correct states. Fully coupled architectures utilize velocity information for attitude correction through the cross-covariance $P_{\theta v}$:

(6) $$\delta \hat{\theta }=K_{\theta }\cdot \Delta v,\;K_{\theta }=P_{\theta v}{\left(P_{vv}+R\right)}^{-1}$$

Undersized correction gains result in insufficient attitude correction. Residual attitude errors easily form prediction-update cycles, leading to cumulative error trends. For maneuvering AUVs, certain attitude error states (such as down-tilt misalignment angles) couple with gyroscope biases in the unobservable subspace [26]. When $P_{\theta v}$ becomes distorted, the correction of these error states becomes further constrained.

Fully coupled architectures face three problems in high-curvature scenarios: high curvature increases $F_{v\theta }$, high dynamics make accurate *Q* modeling difficult, and the fully coupled architecture amplifies $P_{\theta v}$ distortion. Within the coupling mechanism, the coupling term $F_{v\theta }$ amplifies error propagation, while the cross-correction that should be achieved through $P_{\theta v}$ fails due to covariance distortion, with negative effects dominating. Under these circumstances, decoupling attitude from the state vector trades coupling benefits for system robustness, suppressing error drift during high-curvature motion.

## 3. Design Principles for Decoupled Navigation

The decoupled architecture fundamentally eliminates the attitude-velocity error propagation pathway, thereby preventing error amplification during high-curvature motion. However, while decoupling enhances robustness, it introduces new challenges: achieving and maintaining the decoupled state, meeting precision requirements for independent attitude estimation, and compensating for information loss caused by decoupling. This chapter examines these critical issues from a design perspective, establishing the conditions and principles that decoupled navigation schemes must satisfy.

### 3.1. Implementing Decoupling

The core objective of decoupling is to remove attitude from the state vector, i.e., δθ∉x, thereby reducing the navigation filter to a nine-dimensional state space:

(7) $$x={\left[\delta p^{T},\delta v^{T},\delta b_{a}^{T}\right]}^{T}\in R^{9}$$

Attitude serves as an external input rather than an estimated state. During the prediction phase, the coupling term vanishes from the state transition matrix, preventing deterministic propagation of attitude errors to velocity through the state equations. During the update phase, the absence of cross-covariance $P_{\theta v}$ yields a correction gain $K_{\theta }=0$, ensuring velocity observations do not provide feedback correction to attitude. Attitude errors transition from state variables to external disturbances.

The decoupled architecture sacrifices optimality under ideal conditions in exchange for reduced sensitivity to modeling accuracy. By removing attitude errors from the state space, system performance depends primarily on the boundedness of attitude errors rather than the precision of covariance propagation. This design proves more suitable for high-dynamic scenarios where accurate modeling is challenging.

### 3.2. Requirements for Independent Attitude Estimation

Decoupling requires the attitude to be obtained from an independent estimation module that must satisfy multiple conditions to ensure the effectiveness of the decoupled approach.

The fundamental requirement is attitude error boundedness. Bounded attitude error guarantees controllable velocity error:

(8) $$∥\delta v∥\leq ∥F_{v\theta }∥\cdot {\tilde{q}}_{\max}\cdot \Delta t$$

Even when $∥F_{v\theta }∥$ increases, bounded ${\tilde{q}}_{\max}$ ensures controllable velocity error. The periodic resets from DVL measurements prevent accumulation. Achieving boundedness requires absolute observation references. Although tilt estimation is affected by centripetal acceleration, the accelerometer provides a gravity direction reference enabling long-term convergence. Heading estimation relying solely on gyroscopes accumulates drift. However, magnetometers measure the geomagnetic field direction. Since the geomagnetic field $B_{E}$ is independent of vehicle acceleration, magnetometers provide absolute heading constraints. This differs fundamentally from fully coupled architectures that rely on DVL observations to indirectly constrain attitude through the distorted $P_{\theta v}$—absolute references do not depend on covariance propagation.

VQF satisfies the boundedness requirement. Based on complementary filtering principles, VQF decouples tilt and heading processing, utilizing absolute references from accelerometers and magnetometers, respectively, to correct for errors and prevent gyroscope drift accumulation. It adaptively adjusts fusion weights, reducing instantaneous accelerometer weighting during high dynamics to ensure long-term tilt convergence. Magnetometers continuously constrain heading, ensuring overall attitude boundedness. Therefore, our approach employs VQF as the foundational algorithm for the attitude estimation module.

### 3.3. Compensating for Information Loss

Decoupling eliminates cross-covariance $P_{\theta v}$, resulting in information loss. In fully coupled architectures, $P_{\theta v}$ encodes attitude–velocity correlation, enabling the Kalman gain to automatically adjust observation weighting. In IMU/DVL integrated navigation systems, attitude performs coordinate transformation, converting body-frame velocity to the navigation frame. Attitude errors directly affect velocity estimation and updates, necessitating alternative compensation mechanisms.

The core compensation strategy involves transferring attitude uncertainty from the covariance matrix to the noise covariance matrices. Fully coupled architectures propagate attitude uncertainty through $P_{\theta \theta }$ and leverage $P_{\theta v}$ to influence Kalman gains. In decoupled architectures, attitude becomes an external input to the velocity subsystem, with attitude errors equivalent to external disturbances. Parameterizing attitude uncertainty into the process noise *Q* and measurement noise *R* achieves equivalent observation weight adjustment through Kalman gain *K* computation, consistent with the physical interpretation of process and measurement noise.

The compensation scheme estimates attitude uncertainty based on internal statistics from the independent attitude estimator, circumventing the amplification mechanism analyzed previously. Attitude information solely adjusts noise parameters without entering state estimation or forming feedback loops, preserving the robustness of the decoupled architecture.

## 4. VQF-Based Decoupled Navigation System Design

### 4.1. System Architecture

**Figure 1:**
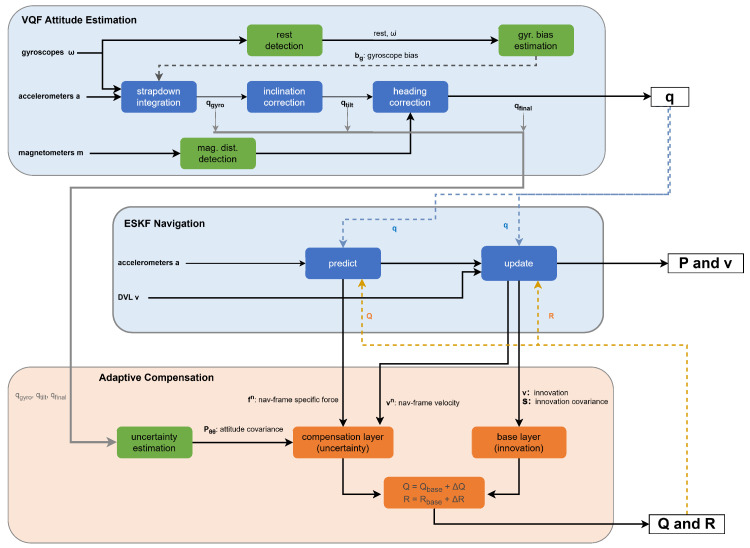
Three-layer system architecture. Top: attitude estimation layer (VQF) outputting quaternion and uncertainty metrics. Middle: 9-state navigation filter. Bottom: adaptive compensation mapping attitude uncertainty to noise parameters. Solid arrows: data flow. Dashed arrows: adaptive feedback.

The system adopts a three-layer architecture comprising the attitude estimation layer, the navigation filtering layer, and the adaptive compensation layer, as illustrated in Figure 1.

The attitude estimation layer operates independently, outputting the attitude quaternion *q*, gyroscope bias $b_{g}$, and attitude quality metrics. The navigation filtering layer employs a state vector that excludes attitude states:

(9) $$x={\left[\delta p^{T},\delta v^{T},\delta b_{a}^{T}\right]}^{T}\in R^{9}$$

The attitude quaternion *q* and gyroscope bias $b_{g}$ serve as external inputs for state updates, while the accelerometer bias is estimated within the navigation layer. The adaptive compensation layer dynamically adjusts the process noise covariance matrix *Q* and measurement noise covariance matrix *R* based on attitude quality metrics.

The attitude and navigation layers employ unidirectional information flow. Since the attitude layer must satisfy boundedness constraints, it utilizes VQF based on complementary filtering principles, whereas the navigation layer implements an adaptive error-state extended Kalman filter (ESKF). The fundamental differences between these frameworks render bidirectional feedback susceptible to iterative interference; unidirectional flow preserves the autonomy of the attitude layer.

Under this unidirectional design, observation information enters each layer according to constraint dimensions, preventing estimation errors from the navigation layer from contaminating attitude estimates through feedback pathways. This layered constraint structure ensures attitude layer independence: even if the navigation layer degrades due to sensor failure, attitude estimation maintains boundedness.

The adaptive compensation layer addresses covariance propagation loss resulting from decoupling. The system implements a dual-layer adaptive compensation mechanism that achieves functional equivalence through explicit adjustment of the Q/R matrices.

### 4.2. VQF Attitude Estimation Module Design

The layered architecture and theoretical analysis demand that attitude estimation support unidirectional information flow, magnetic disturbance robustness, and error observability. Traditional coupled filters exhibit mutual interference between tilt and heading corrections, failing to meet decoupling requirements. Conventional complementary filters lack smooth degradation capabilities or couple magnetometer measurements with tilt estimation. This system employs the VQF algorithm as the foundation for attitude processing. VQF performs independent tilt and heading corrections, supports seamless switching between 9-DoF and 6-DoF IMU modes, and demonstrates superior accuracy and convergence speed compared to existing methods on public datasets.

#### 4.2.1. Input and Processing

The attitude module receives angular velocity ω, acceleration a measurements from the IMU, and magnetometer data, processing them through the VQF algorithm. The three-axis attitude computation proceeds as follows:

1. **Gyroscope integration prediction**: Angular velocity undergoes bias compensation followed by quaternion integration to yield the predicted vehicle attitude.
2. **Almost-inertial frame filtering**: The acceleration measurement transforms to an almost-inertial frame (a slowly drifting quasi-inertial reference). Within this coordinate system, independent second-order Butterworth low-pass filtering applies to each acceleration component, effectively separating gravitational acceleration from linear acceleration components to obtain preprocessed acceleration values.
3. **Tilt constraint**: The extracted gravity direction constrains the attitude prediction for roll and pitch angles, suppressing gyroscope integration drift.
4. **Heading correction**: For yaw angle estimation, VQF employs magnetometer data from the 9-DoF IMU as the heading observation source, implementing heading correction through a first-order exponential filter:
  (10) $$\psi_{k+1}=\left(1-\kappa \right)\psi_{k}^{\mathrm{gyro}}+\kappa \psi_{k}^{\mathrm{mag}}$$
  where the filter gain κ parameterizes through the time constant:
  (11) $$\kappa =1-e^{-\Delta t/\tau_{\mathrm{mag}}}$$

The heading constraint mechanism features scalar angle input, decoupled from tilt correction. Leveraging this characteristic, we abstract heading correction as a generic interface, defining a standardized observation triplet:

(12) $$\left(\psi_{\mathrm{obs}},\sigma_{\psi },\mathrm{valid}\right)$$

where $\psi_{\mathrm{obs}}$ denotes the observed angle, $\sigma_{\psi }$ represents uncertainty, and valid indicates the validity flag. For magnetometers providing absolute constraints, the system directly outputs $\psi_{\mathrm{obs}}=\psi_{\mathrm{mag}}$. For sensors providing relative constraints (e.g., USBL/sonar), a preprocessing layer converts relative measurements to absolute heading: $\psi_{\mathrm{obs}}=\psi_{\mathrm{rel}}+\psi_{\mathrm{init}}$.

When replacing the magnetometer with alternative heading sensors (e.g., USBL, sonar), beyond converting relative constraints to absolute constraints through the preprocessing layer, adjustment of VQF’s time constant parameterization requires consideration. Building upon the original formulation’s sampling-rate-independent implementation, we propose the following adaptation strategy:

1. If a low-frequency sensor (e.g., 1 Hz USBL) exhibits observation quality comparable to magnetometers, $\tau_{\mathrm{mag}}$ may remain unchanged. Under this configuration, the per-update gain κ automatically increases by approximately 100× according to Equation (11), compensating for reduced update frequency.
2. If the replacement sensor exhibits significantly higher noise, $\tau_{\mathrm{mag}}$ should increase to reduce confidence, or scale proportionally by the frequency ratio: $\tau_{\mathrm{new}}=\tau_{\mathrm{mag}}\cdot {\left(f_{\mathrm{mag}}/f_{\mathrm{new}}\right)}^{\alpha }$ where 0.5≤α≤1.0.
3. If the sensor characteristics and system dynamics matching relationship changes, recalculation based on actual noise and response requirements becomes necessary.

#### 4.2.2. Output Interface

Following the processing described above, the attitude module provides the following outputs:

The attitude quaternion *q* and linear acceleration $a_{\mathrm{lin}}$ feed into the navigation layer. These outputs enter the Kalman prediction stage of the navigation layer. The attitude quaternion *q* performs coordinate transformation, converting DVL-measured body-frame velocity and IMU specific force to the navigation frame:

(13) $$v^{n}=C_{b}^{n}\left(q\right)v^{b},\;f^{n}=C_{b}^{n}\left(q\right)f^{b}$$

The transformed quantities $v^{n}$ participate in Kalman updates within the navigation layer and contribute to velocity prediction.

To realize adaptive compensation of attitude uncertainty, the VQF module additionally outputs three attitude quaternions corresponding to successive correction stages in the VQF algorithm:

(1) The pure gyroscope-integrated attitude $q_{\mathrm{gyro}}$ prior to tilt correction;
(2) The attitude $q_{\mathrm{tilt}}$ after tilt correction but before heading correction;
(3) The final attitude $q_{\mathrm{final}}$ following both tilt and heading corrections.

These three quaternions serve as inputs to the compensation layer, enabling extraction of correction magnitudes at each processing step and statistical characterization of attitude uncertainty, subsequently computing incremental process noise ΔQ and incremental measurement noise ΔR.

### 4.3. Attitude Uncertainty Compensation Strategy

This section designs a dual-layer noise covariance adjustment mechanism: the base layer employs innovation-based adaptive methods to reflect overall system uncertainty, while the compensation layer leverages attitude uncertainty for incremental compensation, addressing information loss caused by decoupling.

The uncertainties from both layers may be regarded as approximately independent: base layer innovations originate from the navigation filter internals (the difference between observations and predictions), reflecting overall model fidelity; compensation layer attitude uncertainty derives from independent VQF estimation, reflecting the specific influence of attitude errors on navigation states. Under the assumption of independence, additive superposition applies:

(14) $$Q=Q_{\mathrm{base}}+\Delta Q_{\theta },\;R=R_{\mathrm{base}}+\Delta R_{\theta }$$

#### 4.3.1. Base Layer: Innovation-Based Adaptation

The base layer implements covariance scaling [27], a well-established adaptive filtering technique. By computing windowed statistics of the innovation sequence, the method estimates actual covariance and compares it with theoretical covariance to derive scaling factors that adjust baseline noise covariances. This work adopts a window length of M=50 with scaling factors constrained to the range [0.5, 4.0]. This base layer captures overall system uncertainty and remains independent of whether the architecture is coupled or decoupled.

#### 4.3.2. Compensation Layer: Attitude Uncertainty Compensation

Attitude uncertainty acquisition proceeds as follows. The VQF outputs three key attitude quaternions: pure gyroscope-integrated attitude $q_{\mathrm{gyro}}$, tilt-corrected attitude $q_{\mathrm{tilt}}$, and final attitude $q_{\mathrm{final}}$. Converting these to Euler angles, we extract the correction magnitude for each angle component from its corresponding correction stage:

(15) $$\begin{array}{cc} \delta_{\mathrm{roll},k} & =|\mathrm{roll}\left(q_{\mathrm{tilt},k}\right)-\mathrm{roll}\left(q_{\mathrm{gyro},k}\right)|\\ \delta_{\mathrm{pitch},k} & =|\mathrm{pitch}\left(q_{\mathrm{tilt},k}\right)-\mathrm{pitch}\left(q_{\mathrm{gyro},k}\right)|\\ \delta_{\mathrm{heading},k} & =|\mathrm{yaw}\left(q_{\mathrm{final},k}\right)-\mathrm{yaw}\left(q_{\mathrm{tilt},k}\right)| \end{array}$$

Roll and pitch corrections derive from accelerometer-based tilt correction, while heading derives from magnetometer correction, consistent with VQF’s decoupled architecture. The correction magnitude reflects the discrepancy between gyroscope-predicted and sensor-corrected attitudes. Its standard deviation over a sliding window quantifies temporal consistency—large fluctuations indicate higher uncertainty, analogous to innovation monitoring in Kalman filters.

Through windowed statistics, we compute standard deviations whose magnitudes reflect the statistical uncertainty of attitude estimation:

(16) $$\begin{array}{cc} \sigma_{\mathrm{roll}} & =\sqrt{\frac{1}{N}\sum_{k=1}^{N}{\left(\delta_{\mathrm{roll},k}-\bar{\delta_{\mathrm{roll}}}\right)}^{2}}\\ \sigma_{\mathrm{pitch}} & =\sqrt{\frac{1}{N}\sum_{k=1}^{N}{\left(\delta_{\mathrm{pitch},k}-\bar{\delta_{\mathrm{pitch}}}\right)}^{2}}\\ \sigma_{\mathrm{heading}} & =\sqrt{\frac{1}{N}\sum_{k=1}^{N}{\left(\delta_{\mathrm{heading},k}-\bar{\delta_{\mathrm{heading}}}\right)}^{2}} \end{array}$$

We construct the attitude uncertainty covariance matrix:

(17) $$P_{\theta \theta }=\mathrm{diag}\left(\sigma_{\mathrm{roll}}^{2},\sigma_{\mathrm{pitch}}^{2},\sigma_{\mathrm{heading}}^{2}\right)$$

The diagonal structure assumes uncorrelated errors across axes, consistent with VQF’s independent processing of tilt and heading.

For robustness enhancement, standard deviations undergo threshold limiting (constrained to [0.001, 0.1] rad in this work).

Attitude errors influence velocity estimation through specific force integration. Notably, fully coupled architectures propagate coupling via recursive $P_{\theta v}$ updates, where amplification under high-dynamic conditions causes propagation distortion. The decoupled architecture employs $P_{\theta \theta }$ solely for covariance transformation at the current instant without recursive propagation, fundamentally avoiding amplification mechanisms. Let $f^{n}$ denote the specific force projection in the navigation frame. Attitude error affects velocity through specific force integration: $\delta v={\left[\delta \theta \right]}_{\times }f^{n}\Delta t$, where ${\left[\cdot \right]}_{\times }$ denotes the skew-symmetric matrix operator. Its covariance becomes:

(18) $$\mathrm{Cov}\left(\delta v\right)={\left[f^{n}\right]}_{\times }P_{\theta \theta }{\left[f^{n}\right]}_{\times }^{T}\Delta t^{2}$$

Therefore, the process noise increment for velocity components is:

(19) $$\Delta Q_{vv}=\alpha_{Q}\cdot {\left[f^{n}\right]}_{\times }P_{\theta \theta }{\left[f^{n}\right]}_{\times }^{T}\Delta t^{2}$$

where $\alpha_{Q}$ represents an empirical coefficient (set to 2.0 in this work). This value exceeds unity because VQF corrections partially compensate for errors, so the extracted uncertainty underestimates the true value. Position components adopt $\Delta Q_{pp}=\Delta Q_{vv}\cdot \Delta t^{2}$, following from velocity error integration, while bias components receive treatment through the base layer. The complete formulation becomes:

(20) $$\Delta Q=\left[\begin{array}{ccc} \Delta Q_{pp} & 0 & 0\\ 0 & \Delta Q_{vv} & 0\\ 0 & 0 & 0 \end{array}\right]$$

Attitude errors influence the observation model through DVL velocity transformation: $v^{n}=C_{b}^{n}\left(q\right)v^{b}$. Its covariance becomes:

(21) $$\mathrm{Cov}\left(\delta v^{n}\right)={\left[C_{b}^{n}\left(q\right)v^{b}\right]}_{\times }P_{\theta \theta }{\left[C_{b}^{n}\left(q\right)v^{b}\right]}_{\times }^{T}$$

Therefore, the measurement noise increment is:

(22) $$\Delta R=\alpha_{R}\cdot {\left[C_{b}^{n}\left(q\right)v^{b}\right]}_{\times }P_{\theta \theta }{\left[C_{b}^{n}\left(q\right)v^{b}\right]}_{\times }^{T}$$

where $\alpha_{R}$ denotes an empirical coefficient (set to 1.0 in this work), since DVL measurement characteristics are well-calibrated.

The scaling coefficients $\alpha_{Q}=2.0$ and $\alpha_{R}=1.0$ are empirically determined based on the following considerations: (1) process noise tends to be underestimated in high-dynamic scenarios; thus, $\alpha_{Q}>1$ provides conservative uncertainty characterization; (2) DVL measurement noise characteristics are relatively stable and well-calibrated; thus, $\alpha_{R}=1.0$ maintains fidelity to actual sensor performance. These values were validated on the experimental dataset and demonstrated robust performance across varying curvature conditions.

Key parameter configurations are summarized in Table 1.

### 4.4. Algorithm Pseudocode

To ensure the reproducibility of the research, this section provides pseudocode for the proposed VQF-EKF algorithm (Algorithms 1–6).
**Algorithm 1** VQF attitude estimation module**Require:** Gyroscope $\omega \in R^{3}$, Accelerometer $a\in R^{3}$, Sampling period $T_{s}$**Ensure:** Quaternions: $q_{\mathrm{gyro}}$, $q_{\mathrm{tilt}}$, $q_{\mathrm{final}}$  1:$\omega_{c}\leftarrow \omega -b_{\omega }$  2:$\theta \leftarrow ∥\omega_{c}∥\cdot T_{s}$  3:**if** θ>ϵ **then**  4:    $q_{\Delta }\leftarrow [\cos\left(\theta /2\right),\;(\omega_{c}/∥\omega_{c}{∥)}^{⊤}{\sin\left(\theta /2\right)]}^{⊤}$  5:**else**  6:    $q_{\Delta }\leftarrow {\left[1,\;{\left(\omega_{c}T_{s}/2\right)}^{⊤}\right]}^{⊤}/∥{\left[1,\;{\left(\omega_{c}T_{s}/2\right)}^{⊤}\right]}^{⊤}∥$  7:**end if**  8:$q_{\mathrm{gyro}}\leftarrow q_{\mathrm{gyro}}⊗q_{\Delta }$, normalize  9:$b_{\omega }\leftarrow U_{PDATE}G_{YRO}B_{IAS}\left(\omega ,a,b_{\omega },q_{\mathrm{gyro}}\right)$10:$a^{I}\leftarrow R{\left\{q_{\mathrm{gyro}}\right\}}^{⊤}a$11:${\hat{g}}^{I}\leftarrow -L_{OW}P_{ASS}\left(a^{I}\right)/∥L_{OW}P_{ASS}\left(a^{I}\right)∥$12:$\delta_{\mathrm{tilt}}\leftarrow {\hat{g}}^{I}\times {\left[0,0,-1\right]}^{⊤}$13:$k_{\mathrm{tilt}}\leftarrow A_{DAPTIVE}G_{AIN}(∥a∥,∥\omega_{c}∥,g)$14:$q_{\mathrm{corr}}\leftarrow {\left[1,\;{\left(k_{\mathrm{tilt}}\cdot \delta_{\mathrm{tilt}}/2\right)}^{⊤}\right]}^{⊤}$, normalize15:qEI←qcorr⊗qEI, normalize16:qtilt←qEI⊗qgyro, normalize17:$\delta \leftarrow H_{EADING}C_{ORRECTION}(q_{\mathrm{tilt}},\delta ,\psi_{\mathrm{obs}})$18:$q_{\delta }\leftarrow {\left[\cos\left(\delta /2\right),\;0,\;0,\;\sin\left(\delta /2\right)\right]}^{⊤}$19:$q_{\mathrm{final}}\leftarrow q_{\delta }⊗q_{\mathrm{tilt}}$, normalize20:**return** $q_{\mathrm{gyro}}$, $q_{\mathrm{tilt}}$, $q_{\mathrm{final}}$

**Algorithm 2** Attitude uncertainty quantification
**Require:** $q_{\mathrm{gyro}}$, $q_{\mathrm{tilt}}$, $q_{\mathrm{final}}$, window size *N*, bounds $\left[\sigma_{\min},\sigma_{\max}\right]$**Ensure:** Attitude uncertainty $\Sigma_{\mathrm{att}}\in R^{3\times 3}$  1:

$\left(\phi_{\mathrm{gyro}},\theta_{\mathrm{gyro}},\psi_{\mathrm{gyro}}\right)\leftarrow Q_{UAT}2E_{ULER}\left(q_{\mathrm{gyro}}\right)$

  2:

$\left(\phi_{\mathrm{tilt}},\theta_{\mathrm{tilt}},\psi_{\mathrm{tilt}}\right)\leftarrow Q_{UAT}2E_{ULER}\left(q_{\mathrm{tilt}}\right)$

  3:

$\left(\phi_{\mathrm{final}},\theta_{\mathrm{final}},\psi_{\mathrm{final}}\right)\leftarrow Q_{UAT}2E_{ULER}\left(q_{\mathrm{final}}\right)$

  4:

$\delta_{\phi }\leftarrow A_{NGLE}W_{RAP}\left(\phi_{\mathrm{tilt}}-\phi_{\mathrm{gyro}}\right)$

  5:

$\delta_{\theta }\leftarrow \theta_{\mathrm{tilt}}-\theta_{\mathrm{gyro}}$

  6:

$\delta_{\psi }\leftarrow A_{NGLE}W_{RAP}\left(\psi_{\mathrm{final}}-\psi_{\mathrm{tilt}}\right)$

  7:Update sliding window buffers $B_{\phi }$, $B_{\theta }$, $B_{\psi }$  8:$\sigma_{\phi }^{2}\leftarrow \mathrm{Var}\left(B_{\phi }\right)$, $\sigma_{\theta }^{2}\leftarrow \mathrm{Var}\left(B_{\theta }\right)$, $\sigma_{\psi }^{2}\leftarrow \mathrm{Var}\left(B_{\psi }\right)$  9:Apply bounds: $\sigma_{i}\leftarrow \mathrm{clip}\left(\sqrt{\sigma_{i}^{2}},\sigma_{\min},\sigma_{\max}\right)$10:**return** $\Sigma_{\mathrm{att}}=\mathrm{diag}\left(\sigma_{\phi }^{2},\sigma_{\theta }^{2},\sigma_{\psi }^{2}\right)$


**Algorithm 3** Dual-layer adaptive noise compensation
**Require:** Innovation $\nu_{k-1}$, $S_{k-1}$, specific force $f^{n}$, velocity $v^{n}$, $\Sigma_{\mathrm{att}}$, $T_{s}$**Ensure:** Adapted noise matrices Q, R  1:Update innovation buffer $B_{\nu }$  2:

${\hat{C}}_{\nu }\leftarrow \frac{1}{|B_{\nu }|}\sum_{i}\nu_{i}\nu_{i}^{⊤}$

  3:

$\lambda \leftarrow \mathrm{clip}(\mathrm{tr}\left({\hat{C}}_{\nu }\right)/\mathrm{tr}\left(S_{k-1}\right),\lambda_{\min},\lambda_{\max})$

  4:$Q_{\mathrm{base}}\leftarrow \lambda Q_{0}$, $R_{\mathrm{base}}\leftarrow \lambda R_{0}$  5:

$\Delta Q_{vv}\leftarrow \alpha_{Q}{\left[f^{n}\right]}_{\times }\Sigma_{\mathrm{att}}{\left[f^{n}\right]}_{\times }^{⊤}T_{s}^{2}$

  6:

$\Delta R\leftarrow \alpha_{R}{\left[v^{n}\right]}_{\times }\Sigma_{\mathrm{att}}{\left[v^{n}\right]}_{\times }^{⊤}$

  7:**return** $Q=Q_{\mathrm{base}}+\Delta Q$, $R=R_{\mathrm{base}}+\Delta R$


**Algorithm 4** ESKF prediction
**Require:** Accelerometer a, quaternion q, time step Δt, process noise Q**Ensure:** Predicted state $x^{-}$, covariance $P^{-}$, specific force $f^{n}$  1:

$C_{b}^{n}\leftarrow R{\left\{q\right\}}^{⊤}$

  2:

$f^{n}\leftarrow C_{b}^{n}\left(a-b_{a}\right)$

  3:

$a^{n}\leftarrow f^{n}+{\left[0,0,g\right]}^{⊤}$

  4:

$p\leftarrow p+v\Delta t+\frac{1}{2}a^{n}\Delta t^{2}$

  5:

$v\leftarrow v+a^{n}\Delta t$

  6:

$F\leftarrow \left[\begin{array}{ccc} I_{3} & I_{3}\Delta t & 0\\ 0 & I_{3} & -C_{b}^{n}\Delta t\\ 0 & 0 & I_{3} \end{array}\right]$

  7:

$P^{-}\leftarrow FPF^{⊤}+Q$

  8:**return** $x^{-}={\left[p^{⊤},v^{⊤},b_{a}^{⊤}\right]}^{⊤}$, $P^{-}$, $f^{n}$


**Algorithm 5** ESKF measurement update
**Require:** DVL velocity $v_{\mathrm{dvl}}^{b}$, quaternion q, predicted $x^{-}$, $P^{-}$, noise R**Ensure:** Updated $x^{+}$, $P^{+}$, innovation ν, S  1:

$v_{\mathrm{dvl}}^{n}\leftarrow R{\left\{q\right\}}^{⊤}v_{\mathrm{dvl}}^{b}$

  2:

$H\leftarrow [0_{3\times 3},\;I_{3},\;0_{3\times 3}]$

  3:

$\nu \leftarrow v_{\mathrm{dvl}}^{n}-Hx^{-}$

  4:

$S\leftarrow HP^{-}H^{⊤}+R$

  5:

$K\leftarrow P^{-}H^{⊤}S^{-1}$

  6:

$x^{+}\leftarrow x^{-}+K\nu$

  7:

$P^{+}\leftarrow \left(I-KH\right)P^{-}{\left(I-KH\right)}^{⊤}+KRK^{⊤}$

  8:**return** $x^{+}$, $P^{+}$, ν, S


**Algorithm 6** VQF-EKF integrated navigation system
**Require:** IMU stream at $f_{\mathrm{IMU}}$ Hz, DVL stream at $f_{\mathrm{DVL}}$ Hz  1:Initialize: $x\leftarrow 0_{9}$, $P\leftarrow P_{0}$, $Q\leftarrow Q_{0}$, $R\leftarrow R_{0}$  2:**On IMU callback **$\left(\omega ,a,t_{\mathrm{imu}}\right)$:  3:   $\left(q_{\mathrm{gyro}},q_{\mathrm{tilt}},q\right)\leftarrow VQF\left(\omega ,a,T_{s}\right)$  4:   $\Sigma_{\mathrm{att}}\leftarrow A_{TTITUDE}U_{NCERTAINT}y\left(q_{\mathrm{gyro}},q_{\mathrm{tilt}},q\right)$  5:   $\left(x,P^{-},f^{n}\right)\leftarrow P_{REDICT}\left(a,q,T_{s},Q\right)$  6:   Store $\left(t_{\mathrm{imu}},x,P^{-},q,f^{n},\Sigma_{\mathrm{att}}\right)$ to history buffer H  7:**On DVL callback **$\left(v_{\mathrm{dvl}}^{b},t_{\mathrm{dvl}}\right)$:  8:   $\left(x^{-},P^{-},q,f^{n},\Sigma_{\mathrm{att}}\right)\leftarrow S_{YNCHRONIZE}\left(H,t_{\mathrm{dvl}}\right)$  9:   $v^{n}\leftarrow R{\left\{q\right\}}^{⊤}v_{\mathrm{dvl}}^{b}$10:   $\left(Q,R\right)\leftarrow A_{DAPTIVE}\left(\nu_{\mathrm{prev}},S_{\mathrm{prev}},f^{n},v^{n},\Sigma_{\mathrm{att}},T_{s}\right)$11:   $\left(x^{+},P^{+},\nu ,S\right)\leftarrow U_{PDATE}\left(v_{\mathrm{dvl}}^{b},q,x^{-},P^{-},R\right)$12:   $\nu_{\mathrm{prev}}\leftarrow \nu$, $S_{\mathrm{prev}}\leftarrow S$, $x\leftarrow x^{+}$, $P\leftarrow P^{+}$


The system operates with two asynchronous callbacks. The IMU callback performs VQF attitude estimation and ESKF prediction at high rate (e.g., 100 Hz). The DVL callback performs measurement update at low rate (e.g., 10 Hz) with proper time synchronization. The compensation layer maps attitude uncertainty $\Sigma_{\mathrm{att}}$ to noise increments: $\Delta Q_{vv}=\alpha_{Q}{\left[f^{n}\right]}_{\times }\Sigma_{\mathrm{att}}{\left[f^{n}\right]}_{\times }^{⊤}T_{s}^{2}$ and $\Delta R=\alpha_{R}{\left[v^{n}\right]}_{\times }\Sigma_{\mathrm{att}}{\left[v^{n}\right]}_{\times }^{⊤}$.

## 5. Experiment

This chapter validates the theoretical analysis and algorithmic design presented in previous chapters through real-world IMU/DVL integrated navigation data.

### 5.1. Experimental Data and Analysis Strategy

The experimental dataset comprises authentic IMU/DVL measurements collected from the Danjiangkou Reservoir area in China. The trajectory spans 391.951 m over approximately 650 s and includes multiple curved turning segments with maximum curvature of approximately $0.28\;m^{-1}$ (corresponding to a turning radius of approximately 4 m) and average velocity of approximately 0.6m/s. The AUV platform is equipped with an inertial navigation system and auxiliary sensors, whose primary performance specifications are summarized in Table 2.

The trajectory encompasses high-curvature maneuvering scenarios emphasized in the theoretical analysis, contrasting with conventional straight-line or regular grid paths. It represents typical scenarios where traditional navigation algorithms encounter challenges. The dataset covers a broad range of operating conditions: velocity varies from 0.3 to 0.9 m/s, curvature ranges from 0.04 to 0.28 $m^{-1}$, and the resulting centripetal acceleration spans approximately 0.01 to 0.22 m/$s^{2}$. To demonstrate performance variations under different conditions, the trajectory is manually partitioned into four representative segments based on curvature characteristics for comparative analysis.

Since GPS signals are unavailable underwater, high-precision ground truth is obtained by conducting experiments in shallow water. A GPS antenna is mounted at an elevated position on the vehicle body to ensure continuous GPS signal reception while IMU and DVL operate normally, with the platform navigating at depths of 0.5–2 m. The reference trajectory generation procedure follows these steps:

1. RTKLib (version 2.4.3, developed by the RTKLIB Development Team, Tokyo, Japan) performs differential post-processing on raw GPS observations to obtain centimeter-level positioning results.
2. The final reference trajectory is generated through RTS smoothing, combining high-frequency IMU attitude and DVL velocity information.

Closure error assessment from round-trip segments indicates that the reference trajectory exhibits cumulative position error of approximately 0.8 m (closure error rate of 0.2%), meeting the precision requirements for algorithm performance evaluation.

The VQF-EKF configuration is specified in Table 3.

The AEKF baseline algorithm employs a 16-dimensional error-state filter architecture with parameters configured according to standard underwater navigation settings [28], including IMU noise parameters derived from Allan variance analysis, DVL measurement noise, and innovation-based adaptive strategy (window length of 50 DVL observations, adaptive factor range [0.5, 4.0]). This configuration has been fine-tuned on the experimental trajectory to ensure convergence stability.

**Figure 2:**
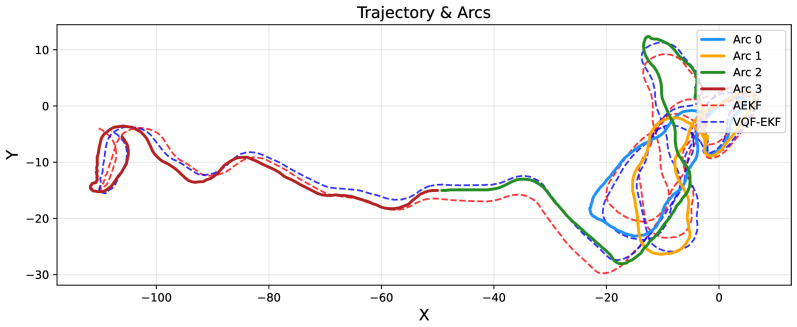
Trajectory comparison.

Figure 2 presents the overall trajectory layout and segmentation results. Solid lines represent ground truth trajectories, while dashed lines denote estimated trajectories (red for AEKF, blue for VQF-EKF). The four analysis segments are marked with distinct colors. In the high-curvature Segment 2 region (the circular segment on the right side of the trajectory), AEKF exhibits significant deviation from ground truth, whereas VQF maintains excellent tracking performance, intuitively validating the advantages of the decoupled architecture in high-curvature scenarios.

### 5.2. Performance Metrics

This study employs the following evaluation metrics:

Root mean square error (RMSE) serves as the primary accuracy indicator. To characterize turning segment performance, particular emphasis is placed on analyzing normal error (deviation perpendicular to the path tangent direction):

(23) $$e_{\mathrm{normal}}=\left(p_{\mathrm{est}}-p_{\mathrm{true}}\right)\cdot n$$

where n denotes the trajectory normal unit vector. According to the analysis in Section 2.1, normal error directly reflects the influence of velocity estimation under high-curvature motion, resulting in lateral deviation, and constitutes a critical metric for evaluating high-curvature segment performance.

Improvement percentage is defined as:

(24) $$\mathrm{Improvement}=\frac{{\mathrm{RMSE}}_{\mathrm{AEKF}}-{\mathrm{RMSE}}_{\mathrm{VQF}}}{{\mathrm{RMSE}}_{\mathrm{AEKF}}}\times 100\%$$

Additionally, the P95 percentile assesses extreme error control capability, while the normal energy ratio:

(25) $$r_{\mathrm{normal}}=\frac{\sum e_{\mathrm{normal}}^{2}}{{\sum |e|}^{2}}$$

which quantifies error directional distribution characteristics. Statistical significance is verified through three methods: paired *t*-test, Wilcoxon signed-rank test, and Bootstrap resampling (1000 iterations, 95% confidence interval).

### 5.3. Full-Trajectory Performance Analysis

Table 4 summarizes the comparative results of the two technical approaches on full-trajectory performance metrics.

VQF-EKF achieves 27.52% improvement in normal RMSE (2.30 m → 1.67 m) and 21.83% improvement in position RMSE (3.12 m → 2.44 m), validating the effectiveness of the decoupled architecture in eliminating attitude–velocity coupling and avoiding lateral deviation accumulation caused by attitude errors.

More significantly, P95 extreme error is substantially compressed by 42.55% (5.0 m → 2.9 m), demonstrating a “peak-suppression” characteristic: the decoupled architecture effectively suppresses error spikes during turning segments. Notably, the normal error median experiences a slight increase of 9.47%, but for safety-critical underwater navigation applications, controlling large errors (>5 m) that may lead to collisions is more critical than optimizing moderate errors, making this tradeoff a reasonable design choice. The 14.02% reduction in normal energy ratio indicates that the adaptive mechanism yields a more balanced error distribution.

**Figure 3:**
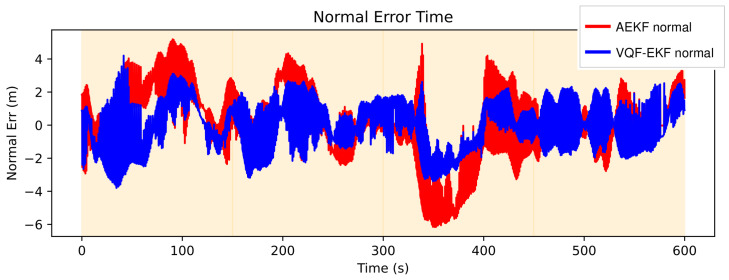
Normal error time series.

Figure 3 illustrates the temporal evolution of normal errors for both algorithms. The VQF-EKF system (blue) not only exhibits a narrower error envelope but, more importantly, avoids AEKF’s loss of control at critical moments. For instance, during the 350–400 s interval, AEKF experiences severe deviation of −6 m, which VQF constrains within −2 m. Improvement is particularly pronounced in the turn-intensive regions near 100 and 300 s, suggesting that algorithmic advantages correlate with motion complexity, consistent with the theoretical analysis in Section 2 regarding error accumulation during high-curvature segments.

Given that this experiment is based on a single trajectory, the following methods are employed to assess improvement stability:

1. **Time series paired test**: Treating the *N* time instants of the single trajectory as *N* paired observations (AEKF error versus VQF error), paired *t*-test, and Wilcoxon signed-rank test are conducted. It should be noted that this method assumes independence of time series points, but temporal autocorrelation exists in practice; therefore, *p*-values serve only as reference indicators. Test results yield p<0.001.
2. **Bootstrap confidence interval**: Block Bootstrap is performed on the trajectory time series (block size = 30 s to preserve temporal correlation), with 1000 resampling iterations. The 95% confidence interval for improvement is [0.593, 0.670], remaining consistently distant from zero, indicating that improvement maintains a consistent direction across different sampling subsets.

**Figure 4:**
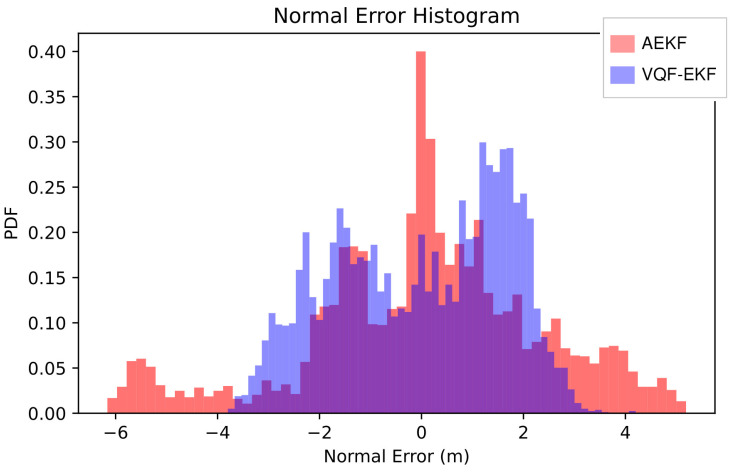
Normal error histogram.

**Figure 5:**
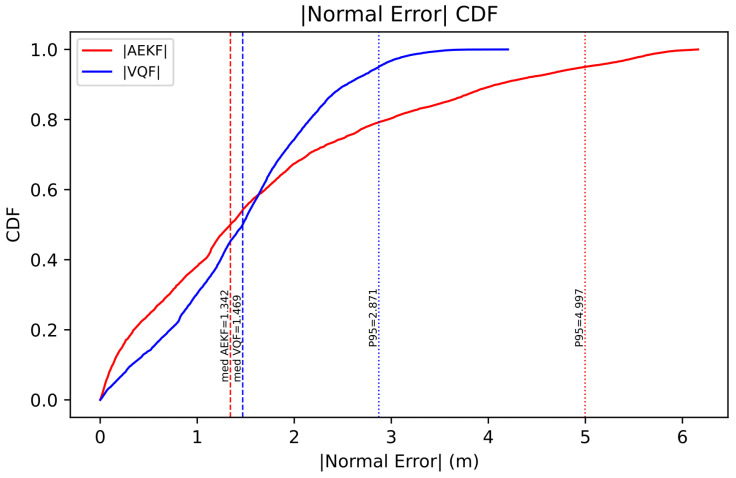
Normal error cumulative distribution function.

Figure 4 and Figure 5 further demonstrate the aforementioned improvement characteristics from a distributional perspective. The histogram reveals that improvement concentrates in the high tail, with AEKF exhibiting a “heavy-tail” morphology while VQF significantly reduces frequency. The cumulative distribution function shows that the VQF curve consistently lies above the AEKF curve. The distributional morphology confirms that improvement derives from tail compression rather than overall shift, embodying a reliability-oriented optimization strategy.

### 5.4. Segment-Level Performance Analysis

Table 5 presents curvature statistics for each segment. It should be noted that the segmentation methodology was determined prior to execution and was not adjusted based on algorithm performance.

**Figure 6:**
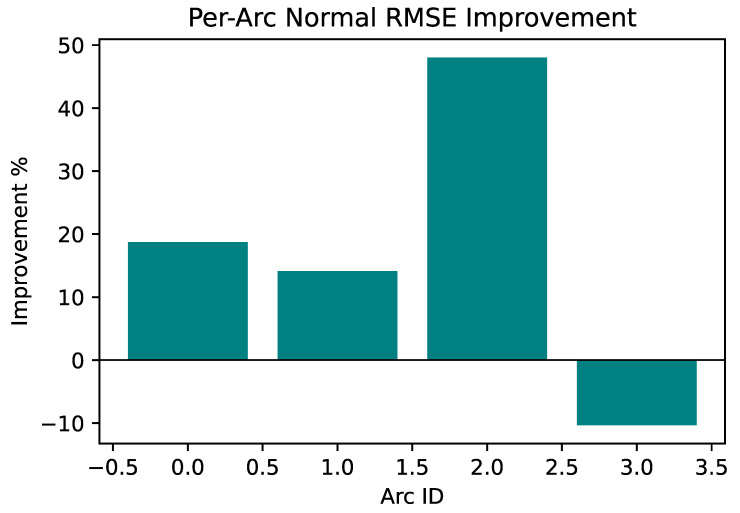
Segment improvement percentage bar chart.

To quantitatively assess the spatial distribution characteristics of improvement, Figure 6 and Table 6 present performance comparisons for each segment.

Results demonstrate significant spatial heterogeneity in improvement. Segment 2 (high-curvature segment) achieves 48.02% improvement, substantially exceeding the 18.72% and 14.13% improvements in Segment 0 and Segment 1. From an absolute error perspective, the high-curvature segment decreases from 3.2487 m to 1.6886 m, yielding an absolute improvement of 1.560 m, constituting the primary contribution source to global improvement.

Segment 3 exhibits a 10.33% relative degradation (absolute error 0.14 m) in the straight-line segment. This represents an architectural tradeoff: under low curvature, VQF’s unidirectional information flow lacks DVL reverse correction, whereas AEKF achieves more robust bidirectional feedback through coupling terms; simultaneously, system parameters are optimized for high-curvature scenarios. This tradeoff is reasonable: sacrificing 0.14 m in a low-risk scenario (baseline 1.34 m) in exchange for 1.56 m improvement in a high-risk scenario (baseline 3.25 m) yields an absolute improvement ratio of 11:1. Despite the relative degradation in this segment, the absolute error (1.48 m) still meets application requirements.

Segment-level analysis reveals the core advantages of the VQF-EKF architecture: achieving maximum improvement in high-curvature, high-error scenarios, effectively reducing navigation risk, and maintaining stability in scenarios already performing well. This differentiated improvement characteristic aligns with practical navigation reliability requirements.

To provide statistical evidence for the theoretical prediction in Section 2.1 that centripetal acceleration amplifies coupling errors, we conducted a stratified analysis focusing on maneuvering segments (κ≥0.06 $m^{-1}$, corresponding to turning radius ≤17 m). Since centripetal acceleration $a_{c}=v^{2}\kappa$ depends on both velocity and curvature, we stratified the data by velocity to isolate the effect of $a_{c}$.

Traditional metrics such as RMSE characterize average performance but may obscure tail behavior. For safety-critical underwater navigation, a single large error can lead to collision or mission failure, making the probability of extreme events more operationally relevant than mean error. We therefore adopt the large error occurrence rate—the proportion of samples exceeding a specified threshold—as a complementary metric that emphasizes tail-risk behavior. The 3 m threshold is selected based on operational requirements: typical torpedo-shaped AUVs have body lengths of 2–5 m, and position errors exceeding this scale pose significant collision risks in confined underwater environments such as pipeline corridors or offshore platform structures [2,3]. Furthermore, this threshold approximates the minimum turning radius in high-curvature maneuvers (approximately 4 m in this experiment), beyond which the vehicle may deviate into obstacle regions.

**Figure 7:**
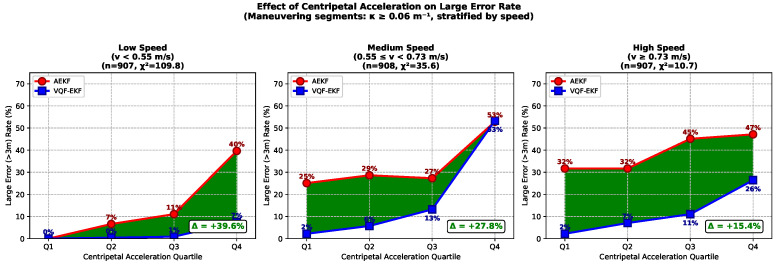
Effect of centripetal acceleration on large error occurrence rate (error >3 m), stratified by velocity. Analysis restricted to maneuvering segments (κ≥0.06 $m^{-1}$). Higher centripetal acceleration consistently increases AEKF large error probability across all speed strata ($\chi^{2}$ tests, all p<0.01), while VQF-EKF maintains lower rates and greater robustness.

As shown in Figure 7, within each speed stratum, data are divided into quartiles by centripetal acceleration (Q1 lowest, Q4 highest). Results demonstrate that AEKF large error occurrence rates increase significantly with centripetal acceleration across all speed strata: from 0% (Q1) to 40% (Q4) in the low-speed stratum ($\chi^{2}=109.8$, p<0.001), from 25% to 53% in the medium-speed stratum ($\chi^{2}=35.6$, p<0.001), and from 32% to 47% in the high-speed stratum ($\chi^{2}=10.7$, p<0.01). This confirms that the probability of large errors correlates significantly with centripetal acceleration, providing statistical support for the theoretical prediction that $a_{c}$ amplifies the coupling term $∥F_{v\theta }∥$.

VQF-EKF demonstrates greater robustness against centripetal acceleration, maintaining substantially lower large error occurrence rates across all conditions. In the low-speed stratum, the performance gap widens from 0% at Q1 to 32% at Q4 (AEKF 40% vs. VQF 7%), indicating that VQF’s advantage becomes more pronounced under high-$a_{c}$ conditions where AEKF suffers severe performance degradation.

It is noted that in the medium-speed stratum at Q4, both methods exhibit similarly high large error rates (approximately 53%). In single-trajectory validation, this velocity-acceleration combination corresponds to a specific sharp turn segment where both methods experience degraded performance due to compound sensor-level factors (e.g., DVL beam geometry variations, transient magnetic disturbances). Since both methods are affected similarly, this does not contradict the main conclusion: VQF’s advantage lies in suppressing coupling-induced error amplification, not in eliminating sensor-level disturbances that affect both architectures. The overall statistical conclusion remains valid ($\chi^{2}$ tests, all p<0.01).

**Figure 8:**
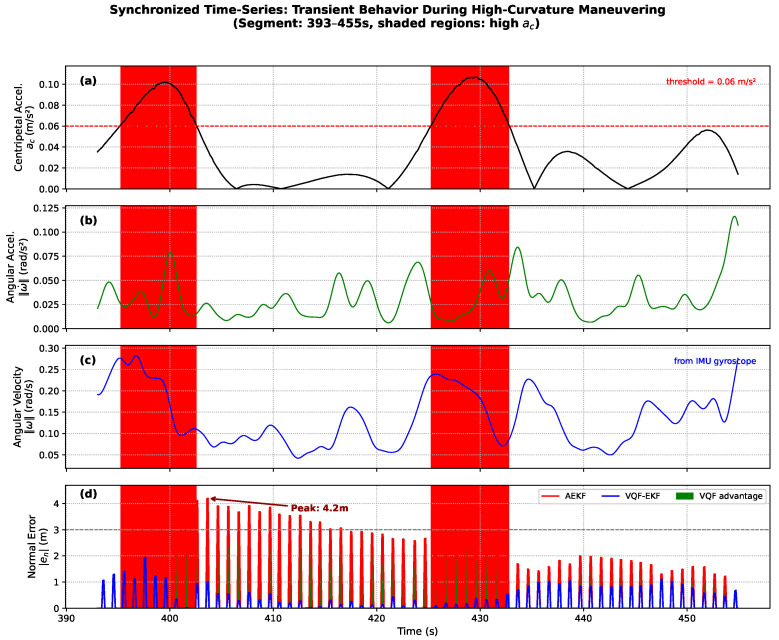
Synchronized time-series during high-curvature maneuvering (393–455 s). (**a**) Centripetal acceleration; (**b**) angular acceleration; (**c**) angular velocity; (**d**) normal error. Shaded regions indicate high-$a_{c}$ periods.

Beyond the statistical evidence, temporal examination provides direct visualization of the relationship between centripetal acceleration and navigation error. Figure 8 presents synchronized time-series for a representative high-curvature segment (393–455 s). The shaded regions indicate periods where $a_{c}>0.06$ m/$s^{2}$. AEKF error peaks (reaching 4.2 m) occur during or immediately following these high-$a_{c}$ intervals, whereas VQF-EKF maintains consistently lower errors throughout.

### 5.5. Experimental Conclusions

This chapter validates the effectiveness of the VQF-EKF hybrid architecture through measured trajectory experiments. Experimental results confirm the theoretical predictions from Section 2 regarding the decoupled architecture’s elimination of coupling errors:

1. For scenarios represented by the experimental data featuring multiple high-curvature maneuvers, global performance improvement is significant, achieving positioning accuracy enhancement compared to the AEKF baseline, validating the decoupled architecture’s effectiveness.
2. Segmentation analysis demonstrates maximum RMSE improvement in high-curvature segments (48% vs. 14–19% in other segments). Stratified centripetal acceleration analysis further reveals that the probability of large errors (>3 m) increases significantly with $a_{c}$ for AEKF ($\chi^{2}$ tests, all p<0.01), while VQF-EKF exhibits greater robustness. These two complementary analyses—one based on average error, the other on tail-risk probability—consistently support the theoretical prediction in Section 2.1 that centripetal acceleration amplifies the coupling term $∥F_{v\theta }∥$.
3. Spatial heterogeneity characteristics indicate that VQF attitude decoupling effectively suppresses coupling errors in high-curvature scenarios, with the adaptive strategy accurately tracking noise variations in dynamic environments; however, the decoupled architecture exhibits adaptability issues in low-curvature scenarios.

Experimental findings provide empirical evidence for in-depth discussion in Section 6 and future research directions.

## 6. Discussion and Conclusions

This research designs a VQF-EKF decoupled architecture that suppresses attitude error propagation to velocity/position through independent attitude estimation. Experimental studies preliminarily demonstrate this strategy’s significant advantages in high-curvature scenarios, validating functional decoupling’s effectiveness in high-dynamic navigation. This result expands the architectural design space for underwater navigation filters, providing diversified architectural options for different scenario requirements.

The spatial heterogeneity characteristics revealed by experiments reflect the complexity and variability of underwater AUV mission scenarios. During high-risk maneuvers such as turning and obstacle avoidance, improvements in navigation accuracy exert the greatest impact on mission success rates, while minor performance differences in straight segments exert limited influence on overall missions. This makes VQF-EKF particularly suitable for high-maneuverability applications such as pipeline inspection, platform circumnavigation, and curved-path manipulation tasks. Simultaneously, segmentation analysis results provide clear directions for subsequent research: adaptive architecture switching based on motion states (decoupled for high curvature, fully coupled for low curvature) may prove more effective than parameter tuning within a single architecture.

This work positions itself as concept validation and characteristic analysis of the VQF-EKF architecture, with the experimental design’s core objective being verification of the theoretical derivation’s prediction that “centripetal acceleration amplifies coupling strength.” To this end, measured trajectories encompassing low, medium, and high curvature modes are selected for segmented verification, with experimental results highly consistent with theory. As the first measured validation of the decoupled architecture, the current work establishes performance baselines and reveals architectural characteristics, laying the foundations for subsequent expansion studies under different sensor configurations, environmental conditions, and voyage lengths. The experimental trajectory provides sufficient diversity for validating the theoretical predictions: velocity ranges from 0.3 to 0.9 m/s and centripetal acceleration spans 0.01 to 0.22 m/$s^{2}$, enabling stratified analysis across different operating conditions. As shown in Figure 7, consistent performance advantages are observed across three velocity strata. The sensor configuration (tactical-grade FOG-IMU with DVL accuracy of ±0.3%) represents typical AUV operational setups. Since the theoretical mechanism that centripetal acceleration amplifies the coupling term $∥F_{v\theta }∥$ is independent of specific sensor grades, the findings are expected to generalize, though validation across different IMU grades (MEMS to navigation-grade) and DVL noise levels remains as future work.

The proposed architecture operates at a different level from algorithmic enhancements such as adaptive IMM or learning-aided methods. These approaches address model uncertainty and residual error compensation on top of a given filter structure, without altering the underlying coupling mechanism. The error amplification pathway analyzed in Section 2 persists regardless of such enhancements. The decoupled architecture addresses this structural issue and can serve as a foundational framework for integrating advanced algorithmic modules. Similarly, loosely coupled variants reduce but do not eliminate cross-covariance propagation, differing fundamentally from complete decoupling with explicit uncertainty compensation.

Future work primarily encompasses two directions:

1. Hybrid adaptive architecture strategy, intelligently switching between decoupled and fully coupled architectures through real-time motion state perception.
2. Improved parameter adaptive mechanisms, exploring intelligent parameter prediction based on deep learning or multi-model switching through motion pattern recognition. Additionally, efforts should advance multi-sensor fusion, long-endurance evaluation, and embedded platform engineering deployment.

## Figures and Tables

**Table 1:** Noise covariance adjustment parameters.

Parameter	Symbol	Value
Innovation window length (base layer)	*M*	50 DVL observations
Scaling factor range	$\left[\lambda_{\min},\;\lambda_{\max}\right]$	[0.5, 4.0]
Correction window length (compensation layer)	*N*	500 steps
Attitude std. dev. limits	$\left[\sigma_{\min},\;\sigma_{\max}\right]$	[0.0001, 0.1] rad
Process noise coefficient	$\alpha_{Q}$	2.0
Measurement noise coefficient	$\alpha_{R}$	1.0

**Table 2:** Sensor specifications.

Sensor	Specification	Value
Fiber Optic Gyroscope	Bias stability	0.3–0.5°/h
	Random walk	0.008–0.015°/$\sqrt{h}$
	Range	±400°/s
	Sampling rate	100 Hz
	Grade	Tactical-grade FOG
Quartz Accelerometer	Bias stability	100–150 μg
	Random walk	40–60 μg/$\sqrt{\mathrm{Hz}}$
	Range	±10 g
	Sampling rate	100 Hz
	Grade	Tactical-grade MEMS
DVL	Velocity accuracy	±0.3% or ±0.5 cm/s
	Range	±10 m/s
	Blind zone	0.5 m
	Sampling rate	10 Hz
	Type	Standard Doppler velocimeter
GPS Receiver	RTK accuracy	±8 mm + 1 ppm (horizontal)
	Positioning mode	Differential post-processing
	Sampling rate	10 Hz
	Type	High-precision RTK

**Table 3:** VQF-EKF parameter configuration.

Category	Parameter	Value
VQF attitude estimation	$\tau_{\mathrm{acc}}$	3 s
	$\tau_{\mathrm{mag}}$	6 s
	$t_{\mathrm{forget}}$	50 s
IMU noise	Gyroscope ARW	<0.015°/$\sqrt{h}$
	Gyroscope bias instability	<0.5°/h
	Accelerometer VRW	<100 μg/$\sqrt{\mathrm{Hz}}$
	Accelerometer bias	<150 μg
Measurement noise	$\sigma_{\mathrm{DVL}}$	0.3%×vs.+5 mm/s
Adaptive EKF	Innovation window *M*	50 DVL observations
	Forgetting factor *b*	0.99
	Scaling factor α	[0.5, 4.0]

**Table 4:** Full-trajectory performance indicators.

Indicator	AEKF	VQF	Improvement (%)
Normal RMSE (m)	2.3009	1.6676	27.52
Position RMSE (m)	3.1202	2.4391	21.83
|Normal error| median (m)	1.3418	1.4688	−9.47
P95 (m)	4.9967	2.8713	42.55
Normal energy ratio	0.5438	0.4675	14.02

**Table 5:** Curvature characteristics of each segment.

Segment	Length (m)	Avg. Curvature ($m^{-1}$)	Max. Curvature ($m^{-1}$)	Curvature Type
Seg0	90.94	0.08	0.18	Low–Medium
Seg1	103.59	0.11	0.20	Medium
Seg2	103.89	0.19	0.28	High
Seg3	93.53	0.04	0.12	Low

**Table 6:** Detailed performance indicators of each segment.

Segment	Length (m)	Proportion (%)	AEKF-RMSE (m)	VQF-RMSE (m)	Improvement (%)
Seg0	90.94	23.2	2.3540	1.9133	18.72
Seg1	103.59	26.4	1.8146	1.5581	14.13
Seg2	103.89	26.5	3.2487	1.6886	48.02
Seg3	93.53	23.9	1.3400	1.4784	−10.33

## Data Availability

The data presented in this study are available on request from the corresponding author.

## Nomenclature

**Category**	**Symbol**	**Description**
**State Variables**	x	State vector
	p, δp	Position and position error [m]
	v, δv	Velocity and velocity error [m/s]
	δθ	Attitude error vector [rad]
	$b_{\omega }$	Gyroscope bias [rad/s]
	$b_{a}$	Accelerometer bias [m/$s^{2}$]
**Quaternions**	q	Attitude quaternion
	$q_{\mathrm{gyro}}$	Gyroscope-integrated quaternion
	$q_{\mathrm{tilt}}$	Tilt-corrected quaternion
	$q_{\mathrm{final}}$	Final attitude quaternion
	$q_{\Delta }$	Incremental rotation quaternion
**Matrices**	F	State transition matrix
	$F_{v\theta }$	Velocity-attitude coupling submatrix
	P, $P_{\theta v}$	Covariance and cross-covariance matrix
	$P_{\theta \theta }$, $\Sigma_{\mathrm{att}}$	Attitude uncertainty covariance
	Q, ΔQ	Process noise covariance and increment
	R, ΔR	Measurement noise covariance and increment
	K	Kalman gain matrix
	H	Observation matrix
	S	Innovation covariance matrix
	$C_{b}^{n}$, R{q}	Body-to-navigation rotation matrix
**Measurements**	ω, $\omega_{c}$	Angular velocity (raw/bias-corrected) [rad/s]
	a	Accelerometer measurement [m/$s^{2}$]
	$v_{\mathrm{dvl}}^{b}$	DVL velocity in body frame [m/s]
	$f^{n}$	Specific force in navigation frame [m/$s^{2}$]
	ν	Innovation vector
**Parameters**	$T_{s}$, Δt	Sampling period/time step [s]
	$\alpha_{Q}$, $\alpha_{R}$	Noise scaling coefficients
	λ	Innovation-based scaling factor
	*M*, *N*	Window lengths (innovation/correction)
	κ	Curvature [$m^{-1}$] or filter gain
	$\tau_{\mathrm{mag}}$	Magnetometer time constant [s]
	H	State history buffer
**Uncertainty**	$\sigma_{\phi }$, $\sigma_{\theta }$, $\sigma_{\psi }$	Attitude standard deviations [rad]
	$\delta_{\phi }$, $\delta_{\theta }$, $\delta_{\psi }$	Correction magnitudes [rad]
	$B_{\phi }$, $B_{\theta }$, $B_{\psi }$	Sliding window buffers
	${\left[\cdot \right]}_{\times }$	Skew-symmetric matrix operator
**Physical**	*g*	Gravitational acceleration [m/$s^{2}$]
	$a_{c}$	Centripetal acceleration [m/$s^{2}$]
	*R*	Turning radius [m]
	ϕ, θ, ψ	Roll, pitch, yaw angles [rad]
**Abbreviations**	AUV	Autonomous Underwater Vehicle
	DVL	Doppler Velocity Log
	ESKF	Error-State Extended Kalman Filter
	IMU	Inertial Measurement Unit
	VQF	Versatile Quaternion-based Filter
	NED	North-East-Down coordinate frame
